# Gene Expression Profiling of Human Monocyte-derived Dendritic Cells – Searching for Molecular Regulators of Tolerogenicity

**DOI:** 10.3389/fimmu.2015.00528

**Published:** 2015-10-19

**Authors:** Katina Schinnerling, Paulina García-González, Juan Carlos Aguillón

**Affiliations:** ^1^Immune Regulation and Tolerance Research Group, Programa Disciplinario de Inmunología, Instituto de Ciencias Biomédicas (ICBM), Facultad de Medicina, Universidad de Chile, Santiago, Chile; ^2^Millennium Institute on Immunology and Immunotherapy (IMII), Santiago, Chile

**Keywords:** tolerogenic dendritic cells, microarray, transcriptome, proteome, signaling

## Abstract

The ability of dendritic cells (DCs) to initiate and modulate antigen-specific immune responses has made them attractive targets for immunotherapy. Since DC research in humans is limited by the scarcity of DC populations in the blood circulation, most of our knowledge about DC biology and function has been obtained *in vitro* from monocyte-derived DCs (moDCs), which can be readily generated in sufficient numbers and are able to differentiate into distinct functional subsets depending on the nature of stimulus. In particular, moDCs with tolerogenic properties (tolDCs) possess great therapeutic potential for the treatment of autoimmune diseases. Several protocols have been developed to generate tolDCs *in vitro*, able to reinstruct auto-reactive T cells and to promote regulatory cells. While ligands and soluble mediators, by which DCs shape immune responses, have been vastly studied, the intracellular pathways and transcriptional regulators that govern tolDC differentiation and function are poorly understood. Whole-genome microarrays and proteomics provide useful strategies to dissect the complex molecular processes that promote tolerogenicity. Only few attempts have been made to understand tolDC biology through a global view on “omics” profiles. So far, the identification of a common regulator of tolerogenicity has been hampered by the fact that each protocol, used for tolDC generation, targets distinct signaling pathways. Here, we review the progress in understanding the transcriptional regulation of moDC differentiation, with a special focus on tolDCs, and highlight candidate molecules that might be associated with DC tolerogenicity.

## Introduction

Dendritic cells (DCs) are professional antigen-presenting cells that direct specific immune responses according to the nature of captured antigens and environmental stimuli (1, 2). They form a heterogeneous group, comprising plasmacytoid DCs, CD1c+ and CD141+ myeloid DCs, originating from a common DC precursor (3), as well as inflammatory DCs, that differentiate from monocytes under inflammatory conditions (4).

Immature DCs continuously sample antigen, but represent poor inducers of immune responses (2). Upon recognition of pathogen- or danger-associated patterns and integration of pro-inflammatory signals from the environment, DCs undergo a maturation process, which enables them to migrate toward lymphoid tissues and initiate antigen-specific T cell responses (5, 6). DCs instruct T cells through the presentation of antigen peptides on major histocompatibility complexes (MHC), co-stimulation, and the secretion of T cell-attracting chemokines and cytokines promoting T cell expansion and differentiation into effector cells with a particular cytokine profile (7). DCs are also able to induce and maintain tolerance to harmless and self-antigens, through deletion of auto-reactive T cells, induction of anergy, and/or generation of regulatory T cells (Tregs) (8–11).

*In vitro*, DCs can be differentiated from human peripheral blood monocytes in the presence of granulocyte-macrophage colony-stimulating factor (GM-CSF) and interleukin (IL)-4 (12). Human myeloid and plasmacytoid DC subsets can be obtained from CD34+ cord blood progenitors in stromal cell-containing cultures, supplemented with Flt3L, stem cell factor, and GM-CSF (13). Here, we focus on monocyte-derived DCs (moDCs), which are closely related to inflammatory DCs (4), and represent the model of choice for studies on human DC biology and function, and the design of cell-based immunotherapies targeting antigen-specific immune responses (14–17). Autologous moDCs can be easily obtained in sufficient numbers from peripheral blood of patients, and either matured/activated to induce immunogenic properties (15), or modulated to promote immunoregulatory functions (18, 19).

Several protocols have been developed for the *in vitro* generation of human moDCs with tolerogenic properties (tolDCs), able to silence or reprogram auto-reactive T cells and induce regulatory lymphocytes (18, 20, 21). Their immunoregulatory function has been corroborated *in vivo* in rodent models of autoimmune diseases (22–26), and first clinical trials using autoantigen-pulsed tolDCs in patients with autoimmune disorders confirmed their safety and efficacy (27, 28). Studies on the mechanisms, by which tolDCs modulate T cell responses, indicate that cell-contact via co-stimulatory/co-inhibitory signals (29), and anti-inflammatory cytokines, such as IL-10 and TGF-β (30) are required for tolerance induction. However, the intracellular molecular processes that govern DC differentiation toward a tolerogenic profile are scarcely understood (31).

Microarray technology allows the exploration of genome-wide changes during DC differentiation, maturation, and modulation (32–34). In some studies, microarray data are complemented by proteome-based strategies such as two-dimensional difference gel electrophoresis (2D-DIGE) and mass spectrometry (35–37).

Here, we review recent findings in gene expression analysis of moDCs, with special focus on approaches to unveil the molecular basis of DC tolerogenicity.

## Transcriptional Changes during Dendritic Cell Differentiation from Monocytes

Gene expression studies of monocytes and moDCs revealed that differentiation of monocytes into DCs leads to the downregulation of genes encoding monocyte markers like CD14 and CD163, genes associated with cell adhesion and motility (E-cadherin, galectin-2, PECAM1/CD31, ICAM1/CD54), and signal transduction/growth control (JAK3, GBP2, DUSP6, MAP3K8), as well as genes encoding the chemokines CXCL8/IL-8, CXCL3/MIP-2β, and CCL4/MIP-1β, the cytokines and cytokine receptors tumor necrosis factor (TNF)-α, IL-15, IL-6, IL-6R, IL13RA1, IL10RA, and TGFBR3, and the transcriptional regulators IRF7A, ERF2, FOSB, KLF9, GATA2, JUNB, and NFKBIA (32, 35, 36). By contrast, genes encoding proteins related to pattern recognition and antigen uptake (MRC1, FcγRII/CD32, NOD1), antigen processing and presentation (LAMP1, HLA-DPA1, HLA-DQA2, CD1a), and co-stimulation (CD83, CD86) were upregulated, together with genes encoding growth factors (TGF-β1, CSF1), cytokines and their receptors (IL-1β, TNFR2, IL1R1), lymphocyte attracting chemokines (CCL13/MCP-4, CCL17/TARC, CCL18/PARC, CCL22/MDC) and chemokine receptors (CCR5, CCRL2), enzymes and carriers of lipid metabolism (ALOX15, LIPA, CYP27A1), adenosine receptors (ADORA1, ADORA2B), signaling molecules (RAP1GAP, IP3KB, TRAF3), and transcription factors IRF4, C/EBP–α, PPAR-γ, p53, and c-myc (32, 35, 36).

At protein level, the chaperones HSP27 and GRP78, as well as proteins involved in Ca2+-binding (S100A9/MRP14, S100A8/MRP8), fatty acid binding (FABP4, FABP5, acyl-CoA-binding protein), cell signaling (GNAI2, ANXA2), oxidative stress (PRDX3, SOD2), and cell structure (vimentin) were found to be upregulated in DCs (35, 36).

## Changes in Gene Expression Profiles upon Dendritic Cell Maturation

Several stimuli are used to induce maturation of DCs *in vitro*, including pro-inflammatory cytokines and microbial products, leading to morphological changes, upregulation of MHC, and co-stimulatory molecules, as well as characteristic chemokine and cytokine profiles (38–42).

Gene expression studies confirmed that previously described markers of mature DCs, such as the co-stimulatory/co-activating molecules CD86, CD83, and CD40, the cell adhesion molecules ICAM1/CD54 and CD49d, the lymph node homing-mediating chemokine receptors CCR7 and CXCR4, and the pro-­inflammatory cytokines TNF-α, IL-1β, and IL-6 were upregulated at transcriptional level, too, regardless of whether maturation was induced by cytokines or pathogen-derived stimuli (38–42). Similarly, transcriptome studies revealed a characteristic chemokine pattern in mature DCs, including the upregulation of CCL2/MIP-1α, CCL8/PARC, CCL17/TARC, CCL22/MDC, CXCL8/IL-8, CXCL9/MIG, CXCL10/IP-10, and CXCL11/I-TAC transcripts (38–42). The global view on gene expression profiles uncovered also differences in transcriptional patterns of moDCs matured with distinct stimuli, despite comparable morphology and phenotype (43). For example, TNF-α-matured DCs exhibit a transcriptional profile similar to immature DCs, characterized by the upregulation of transcripts associated with pattern recognition and phagocytosis (CD209, CD205, FCGRIIB, FCAR, FCER2, C1QA), cell adhesion (CD97, integrin β2/CD18, CD11b), transcriptional regulation (NFKBIA, EGR1), and tryptophan catabolism (IDO) (35, 36). Interferon (IFN)-α-matured DCs, in contrast, display an upregulation of genes encoding maturation-associated proteins (several HLA molecules, LAMP3), transcription factors of the IFN pathway (STAT1, IRF7), components of the antiviral response (PKR, Mx1, TRAIL, granzyme, caspase 1), as well as proteins related to TLR signaling (TLR2, TLR3, MyD88) (43, 44).

Oligonucleotide microarrays of human moDCs, matured by the exposure to bacteria, fungi, viruses, or their components, revealed not only pathogen-specific maturation programs but also a common core response to all pathogens (45, 46). This core response comprises the downregulation of genes encoding pathogen recognition and phagocytosis receptors (MMR, AP2M1), and the upregulation of genes involved in antigen processing and presentation (HLA, LMP2, TAP1, TAP2), signaling (MyD88, lyn), and migration (fascin), as well as those encoding transcription factors (IRF1, IRF7, STAT1), chemokines required for the recruitment of innate immune cells (CCL3/MIP-1α, CCL4/MIP-1β, CCL5/RANTES), and molecules involved in the killing of invasive microorganisms (SOD2, thioredoxin) (45, 46). Serial analysis of gene expression (SAGE) in lipopolysaccharide (LPS)-matured DCs vs. immature DCs additionally revealed an upregulation of genes encoding the chemokines CCL18/PARC and CCL19/MIP-3β/ELC, LAMP3, related to antigen processing and presentation, as well as genes associated with cytokine signaling pathways (IL27B/EBI3, IFI27, ISG20), and protein serine/threonine kinase activity (MAP4K3, STK4) (47).

Kinetic analysis of global gene expression during human DC maturation, induced by bacterial lipopolysaccharide (LPS) and IFN-γ, or CD40 ligation, revealed a temporally coordinated transcriptional program: transcripts encoding pro-inflammatory cytokines and chemokines that guide immune cells to the sites of inflammation (CCL4/MIP-1β, CXCL2/MIP-2α) were early induced upon maturation, followed by an increase of transcripts encoding T cell-attracting chemokines (CCL5/RANTES, CCL15/MIP-1δ), and late upregulation of genes related to survival (CLU, IAP-C, GADD45A), lysosomal function (LAMP3) and response to chemical stimuli (MT1E, MT1G) (33, 48). By contrast, genes encoding the aforementioned maturation markers, proteins involved in antigen processing and presentation (MHCI, TAP1, TAP2), the transcription factors IRF4 and IRF8, and the oxidative stress-associated molecules SOD2 and MT2A were upregulated at a constant level throughout maturation (33, 48).

Maturation induced by a standard cytokine cocktail containing TNF-α, IL-1β, prostaglandin E2 (PGE2) and IL-6, or an alternative cocktail, containing TNF-α, IL-1β, IFN-α, IFN-γ, and poly (I:C), increases the transcription of the co-inhibitor PD-L1, cell adhesion molecules (LFA3/CD58, PSGL1/CD162), cytokine receptors (IL-6Rβ/gp130, IL-2Rγ/CD132, IL4RA/CD124, IL7RA/CD127, IL15RA), transcriptional regulators (RelB, NFKBIA, IRF1, RUNX3), apoptosis regulators (TNFAIP3, TNFAIP6, CFLAR), and enzymes SOD2 and IDO (49, 50). An integrated transcriptomic and proteomic analysis of cytokine-matured DCs identified five major pathways that were differentially regulated during DC maturation, at both RNA and protein levels, comprising cell adhesion, TLR signaling, PPAR signaling and lipid metabolism (PIK3R1, ACSL4, GK, DBI), migration, and cytokine-cytokine receptor interaction (CSF2RA, PTK2B), accompanied by the upregulation of transcription factors NFKB1, NFKB2, and RELA (37).

## Searching Molecular Regulators of Dendritic Cell Tolerogenicity

### Generation of Human Tolerogenic Dendritic Cells

Several protocols have been established to obtain human tolDCs with stable tolerogenic features from peripheral blood monocytes, differing in culture duration and nature of modulating agents. Common strategies are the modulation with anti-inflammatory cytokines, such as IL-10 (51) or TGF-β (52), immunosuppressive drugs, including dexamethasone (53), rapamycin (54), aspirin (55), the PPAR-γ inhibitor rosiglitazone (56), tacrolimus (57), and the JAK inhibitor tofacitinib (58); natural compounds such as resveratrol (59), curcumine (60), 9-cis-retinoic acid (56), 1,25-dihydroxyvitamin D3 (vitD3), either alone (61) or in combination with dexamethasone (62); the HO-1 inducer cobalt protoporphyrin (63), and the NF-κB inhibitor BAY11-7082 (27).

Alternative or partial activation of DCs has been considered as essential for the efficacy of tolDC-based immunotherapy and can be achieved by adding LPS (10, 64, 65), its non-toxic analog monophosphoryl lipid A (66), CD40L (66), or the standard cytokine cocktail for DC maturation (67). This endows tolDCs with enhanced IL-10 production, antigen presentation, and lymph node homing capacity, while preserving a stable tolerogenic profile upon exposure to activating stimuli (68).

Despite the diversity of stimuli used to obtain tolDCs, and although some properties vary amongst protocols, there is a consensus about fundamental features that tolDCs must possess, including low expression of co-stimulatory molecules, high production of anti-inflammatory cytokines, mainly IL-10, and low levels of pro-inflammatory cytokines, as well as the ability to induce T cell hyporesponsiveness or Tregs (67, 69, 70).

### Global Gene Expression Profiling of Tolerogenic Dendritic Cells

To date, few studies have attempted to unravel the molecular basis of DC tolerogenicity through transcriptome and proteome profiling (Table 1).

**Table 1:** **Upregulated genes and proteins in human tolerized DCs**.

Stimulus	Technique	Functional categories	Upregulated genes or proteins	Reference
**Tolerogenic DCs**				
IL-10	Microarray vs. immature DCs	Defense response/immune response	*CD37, IL8, CXCL1, FCGR2B, IL7, IL7R, CTSB, CTSL, CST3, HLADOB, C2, PLAUR*	(69, 71)
		Lymphocyte activation	*IL7, IL7R, IL4RA, PBEF*	
		Signaling	*TFGB, SMAD3, *ID4*, FSHR, FZD7, FZD2, VCAN, *VDR*, *RELB**	
		Cell adhesion	*THBS1, SPARC, HAPLN1, HAS1, EFEMP2*	
		Metabolism	*TBXA2R, PTGDS, LYPLA3, ADHD4, LENG4, PLTP, RBP4, CHSY1, SIAT4A, GK, NUPL1*	
		Stress response	*SOD2, HSP70*	
		Metal ion binding	*FTH1, LTF, ENPP2, *GLI2*, CD71*	
		Transcription	*KLF2, TRRAP, TCF15, DNMT3B, HIRA, FOXB1, SCAND1, DTX1*	
TGF-β + IL-10	Microarray vs. immature DCs	Defense response/immune response	*CD37, IL8, CXCL1, ENTPD, CTSB, CTSL, CST3, HLADOB, C2, C1QA*	(69)
		Signaling	*TFGB, SMAD3, FSHR, FZD7*	
		Cell adhesion	*THBS1, SPARC, HAPLN1, HAS1, EFEMP2*	
		Metabolism	*TBXA2R, APOA4, PTGDS, LASS4, RBP4*	
		Stress response	*SOD2*	
		Metal ion binding	*FTH1, LTF*	
		Transcription	*TRRAP, DNMT3B, *HIRA*, *SCAND1*, DTX1, *HOXB5*, RBM9*	
IL-6 + IL-10	Microarray vs. immature DCs	Defense response/immune response	*CD37, IL8, CXCL1, FCGR2B, CTSB, C2, CTSL, CST3, HLADOB, C1QA, F13A1, PLAUR*	(69)
		Lymphocyte activation	*IL7R*	
		Signaling	*TGFB, SMAD3, FSHR, FZD7, *RELB**	
		Cell adhesion	*THBS1, SPARC, HAPLN1, HAS1, EGFR EFEMP2*	
		Metabolism	*TBXA2R, APOA4, PRKAG1, LYPLA3, ABHD4, LASS4, RBP4*	
		Stress response	*SOD2*	
		Metal ion binding	*FTH1, LTF, *GLI2**	
		Transcription	**KLF2*, TRRAP, *HOXB5*, *TCF15*, *FOXB1*, DNMT3B, RBM9, *SCAND1*, *HIRA*, DTX1*	
Dexamethasone	DIGE and label-free mass spectrometry vs. immature DCs	Defense response/immune response	C1QB, C1QC, F13A, CATC	(72)
		Signaling	STAB1, OSTF1, TPP1, CLIC2, MRC1	
		Metabolism	FKBP5, ANXA1, IMPDH2	
		Stress response	GPX1	
TX527 (vitD3 analog)	2D-DIGE and MALDI-TOF/TOF vs. immature DCs	Defense response/immune response	NCF2, IL1RN	(73)
		Signaling	EFHD2, ANXA2, EHD4	
		Metabolism	CA2, FBP1, G6PD, ACO1, AKR7A2, AKR7A1, ECHS1, LDHB, TGM2, ACOT7, IDH3B, MGLL, NAMPT	
		Stress response	PDCD6IP	
		Cytoskeleton/cell growth	LSP1, TUBB4, TUBB5, LMNA, FSCN1 CAP1, RhoGDI	
		Protein biosynthesis/proteolysis	CTSD, SERPINB6, CCT1, CACYBP, IF4H EEF1G, EEF2, TUFM, HSP90B1, EIF3S3	
vitD3	Microarray vs. early DCs	Defense response/immune response	*IL1RN, CCL22, CD14*	(34)
		Metabolism	*CA2, GLU3, HK3, PFKFB4, PIK3CG, *CMYC*, PDHA1, AMPK, LDHA, ACC, FBP1*	
		Signaling	**NFKB2*, *RELB**	
		Transcription	*PRR5, PDK4, CEBP*	
		Oxidation-reduction	*ATP5A1, SOD2*	
Dexamethasone + vitD3	Microarray vs. immature DC and LPS-matured DCs	Metabolism	*ACADM, ACADVL, ACO2, ACO2, ACOX2, ACSS1, ALDH2, DHRS9, GAPDH, IDH3A, IDH3B, LDHB, MDH2, ME1, ME3, PCK2, PKM2, SLC27A5, SUCLG1, SUCLG2, TPI1*	(74)
		Oxidation–reduction	*SDHA, ATP5G3, ATP5J, ATP5O, COX6A1, COX7A2, COX11, CYC1, NDUFS1, NDUFS8, NDUFB9, PDHA1, PRDX3, SNCA, UQCRB, UQCRC11*	
		Signaling	*EIF3B, EIF3C/EIF3CL, EIF4A3, PIK3R1, RPS19, RPS21, RPS6KA1, RPS6KA2, NOS3, RPS12, SLC2A5, SLC2A1, PIK3R1*	
		Transcription	**TP53*, TCEB1*	
**Activated tolerogenic dendritic cells**				
IL-10 + LPS	Microarray vs. immature DCs and LPS-matured DCs	Defense response/immune response	*CCL19, CXCL13, TNFR2, DR6, FCGR1A, CASP5*	(71)
		Signaling	*JAK1, RHO6, ITPKC, RGS16, ACPP, MUC1*	
		Cell adhesion	*ITGB3*	
		Metabolism	*GK, CHSY1, BMP2, CHI3L2, NNMT, PAM, ASM3A, MAOA*	
		Metal ion binding	*CD71, ENPP2, SCL31A2*	
		Transcription	**VAV1*, *ARNT2*, *CEBPD*, *FOXO3**	
TX527 (vitD3 analog) + LPS + IFNγ	2D-DIGE and MALDI-TOF/TOF vs. LPS + IFN-γ-matured DCs	Defense response/immune response	NCF2, ANXA6, PSME2, SERPINB9	(73)
		Signaling	EFHD2, GDI1, PPP2R1A, SUMF2, ANXA2, SDCBP	
		Metabolism	CA2, G6PD, FBP1, PCK2, PKM2, IDH3A, ACO2, ACOX1, CES1, TGM2, GM2A, GANAB, OGDH, HADHA, PRDX3, DLD, ACADVL	
		Stress response	ORP150, LTA4H, TXNDC4	
		Cytoskeleton/cell growth	ACTB, ACTG1, ACTR2, ARHGDI1, FSCN1, IMMT, LASP1, LCP1, PHB, TWF2, VIM, WDR1	
		Protein biosynthesis/proteolysis	CTSD, HSPD1, HSPH1, LAP3, SERPINB6, CTSS	
		Oxidation–reduction	ATP5A1, SOD2	
Dexamethasone + CD40L	2D-DIGE and MALDI-TOF/TOF vs. CD40L-matured DCs	Defense response/immune response	IL1RN, SAMHD1	(75)
		Signaling	HNRNPK, DPYSL2	
		Metabolism	FAH, GALK1, GLO1, PPA1, ECHS1, TPII, GSTO1, GSTP1, G6PD, PKM2, ENO1, ACO2, PKM1, ENO3, FTH1, PRDX6, MDH1, IDH1	
		Stress response	HSPA1A, HSPA1B, HSPA8, STIP1	
		Transcription	HNRNPL, EBP1	
		Cytoskeleton/cell growth	ACTB, GSN, LCP1, TUBA1A, ACTB, FSCN1, TUBB, TBCB, TWF2	
		Protein biosynthesis/proteolysis	PSMD13, CTSB, CTSZ, EIF3I, WARS, YARS	
		Oxidation-reduction	GLUD1, SOD2, PRDX4	
vitD3 + CD40L	2D-DIGE and MALDI-TOF/TOF vs. CD40L-matured DCs	Defense response/immune response	IL1RN	(75)
		Signaling	DPYSL2, GRB2	
		Metabolism	CA2, ALDH2, G6PD, GLO1, PGM1, PPA1, ECHS1, TPII, FBP1, PCK2, GSTO1, ENO1, PDHA1, PKM2, ALDOA, PGAM1, AKR1A1, LHDB, FTH1, FTL, GPD2, TKT, TALDO1, DLST, IDH3A, MDH1, ACO2, CS	
		Stress response	HSPA1A, HSPA1B, HSPA8, HYOU1, STIP1	
		Transcription	EBP1	
		Cytoskeleton/cell growth	ACTB, CAPZA1, GSN, GMFG, LCP1, ARHGDIB, TUBA1A, ACTB, FSCN1, ARHGDIA, TWF2	
		Protein biosynthesis/proteolysis	PSMD13, RPLP0, LAP3, WARS, UCHL5, PSMC5, CTSD, CTSH, LAP, TGM2, PDXK	
		Oxidation-reduction	GLUD1, SOD2, CAT, PDIA4	
Dexamethasone + vitD3 + CD40L	2D-DIGE and MALDI-TOF/TOF vs. CD40L-matured DCs	Defense response/immune response	IL1RN, PSMA1, ANXA11, SAMHD1	(75)
		Signaling	HNRNPK, GRB2	
		Metabolism	FAH, ALDH2, G6PD, GALK1, GLO1, PGM1, ESD, PPA1, ECHS1, TPII, FBP1, PCK2, GSTO1, PDHA1, PKM2, ENO1, ALDOA, PGAM1, AKR1A1, IDH1, LHDB, FTH1, FTL, UROD, DDAH2, DLST, ALOX15, PRDX6, IDH3A, MDH1, ACO2	
		Stress response	HSPA1A, HSPA1B, HSPA8, STIP1	
		Transcription	EBP1	
		cytoskeleton/cell growth	ACTB, CORO1A, GSN, CAPZA1, FSCN1, ARHGDIA, ARHGDIB, TWF2	
		protein biosynthesis/proteolysis	PSMD13, PSMD7, TUFM, EEF2, CTSB, CTSD, CTSH, CTSZ, LAP3, PSMA5, TGM2, PDXK, WARS, PEPD	
		oxidation/reduction	GLUD1, CAT, PRDX4, PDIA4	
Dexamethasone + vitD3 + LPS	Microarray vs. LPS-matured DCs	Oxidation–reduction	*ATP5B, ATP5D, ATP5H, ATP5L, BACE1, COX6B1, COX7B, COX8A, COX15, GLRX2, GSR, LHPP, NDUFA3, NDUFA6, NDUFA8, NDUFA12, NDUFB5, NDUFB6, NDUFV3, PINK1, PSENEN, SDHB, TRAK1, UQCR10*,	(74)
		Metabolism	*ACAD8, ACSL5, ALDH3A2, ALDH7A1, ALDH9A1, ALDOA, CYP1B1, DHRS4, DHRS9, ECI2, FBP1, GAPDH, GBA2, GPI, HADHA, HADHB, HK1, HK3, HPSE, MTAP, PKM2, UGDH*,	
		Signaling	*ADRB2, ATM, CHRNB4, EGLN1, EIF3E, EIF3M, EIF4A1, EIF4, EBP1, MAPK14, MAP2K3, MLYCD, MRAS, MTOR, NAA10, PDGFC, PIK3R6, PPAT, PPM1A, P4HTM, PPP2R3A, PRKAR1B, PRKCB, PRKD3, RHOT1, RPS6KA2, RPS6KA3, SLC2A3*	
		Transcription	*APEX1, COP9, COPS5, KAT2B, TCEB1*	

*Genes are displayed in italic and transcription factors are underlined*.

Transcriptome analysis of human tolDCs, obtained by modulation with IL-10 alone or in combination with TGF-β or IL-6, and compared to LPS-matured DCs, revealed an upregulation of 36 common genes in all three tolDC types, belonging to the functional categories of defense response (CD37, CXCL8/IL-8, CXCL1), antigen processing and presentation (CTSB, CTSL, HLA-DOB), TGF-β signaling (TGFB, SMAD3), cell adhesion (THBS1), complement and coagulation cascades (C2), transcription (HOXB5, TRRAP), and lipid metabolism (TBXA2R), while 34 genes were downregulated, including CD48, IL-1A, CCL17/TARC, CD74, CREM, and PRDX5 (69). Upregulation of ENTPD1/CD39 and TRAF6 was specific to IL-10 + TGF-β-treated tolDCs, while the transcription factor ID4 was exclusively upregulated in IL-10-modulated tolDCs (69).

Global gene expression profiles of DCs, treated with IL-10, LPS, or a combination of both, unveiled three functional groups of genes that were regulated by IL-10 alone or in concert with LPS: inhibition of specific immunity and inflammation, tuning of cytokine receptor and G protein-coupled receptor (GPCR) signaling, and stimulation of B cell development/function and lymphoid tissue regeneration (71). Compared to LPS, IL-10 alone induced a limited set of genes, encoding proteins related to B cell differentiation and function (SLAM, IL-7, IL-4Rα, PBEF), GPCR signaling (FZD2), and extracellular matrix (versican). In combination with LPS, IL-10 suppressed the expression of the LPS-inducible genes *CD86, CD83*, *IL12*, and *CCR7*. However, a set of genes was uniquely regulated by simultaneous treatment with IL-10 and LPS, including transcripts of intracellular signal transduction molecules (*RGS16, JAK1*), transcription factors (*CEBP, ARNT2, FOXO3*), and lymphocyte attracting chemokines (*CXCL13/BLC, CCL19*) (71).

Ferreira and colleagues explored global molecular changes induced in human moDCs by vitD3 and its analog TX527 through transcriptomic and proteomic approaches, and assigned differentially regulated genes to three functional categories: cytoskeleton structure, protein biosynthesis/proteolysis, and metabolism (73, 75). VitD3 and TX527 reduced the expression of most cytoskeleton proteins, such as fascin, while enhancing the expression of metabolic proteins, e.g., CA2 and FBP1 (73, 75). Protein proteolysis/biosynthesis comprised the main group of proteins that were upregulated in response to TX527, involving translation (eEF1G, eEF2, EIF3S3, EIF4H) and the MHCI/II pathway, particularly CTSD and CTSS, which mediate the degradation of MHCII invariant chain/CD74 (73). Additionally, TX527 treatment increased the expression of stress response proteins, including SOD2, ORP150, HSPD1, and TXNDC4, and proteins of the cellular defense response, such as LTB4 and NCF2 (73).

The comparison of protein profiles of tolDCs, modulated with vitD3, dexamethasone, or both, and subsequently activated by CD40L, revealed common functional groups that were regulated in all three tolDC types, but not in CD40L-matured DCs (75). These comprised lipid metabolism, i.e., fatty acid oxidation and elongation in mitochondria, glycerophospholipid metabolism and phospholipid degradation, as well as NRF2-mediated oxidative stress response (75). Protein interaction networks and pathway analysis indicated that vitD3, rather than dexamethasone, has a strong impact on metabolic pathways involving lipids, glucose, and oxidative phosphorylation, as well as on mitochondrial processes, including alterations of the mitochondrial transmembrane potential (75). By contrast, dexamethasone was shown to affect predominantly proteins of the stress response, e.g., HSP71, and induced proteins of glutathione metabolism, acute phase response signaling, and MHCII antigen presentation pathways, including multiple isoforms of CTSB, CTSD, and CTSZ (75). Combined treatment with vitD3 and dexamethasone, which promotes a strong tolerogenic profile regarding the modulation of T cell responses (75), induced a unique protein signature, dominated by the metabolic effect of vitD3 (75). When compared to treatment with each stimulus alone, combination of vitD3 and dexamethasone upregulated proteins involved in the anti-apoptotic response (HSPA9, PYCARD, and ANXA1), protein biosynthesis/proteolysis, protein binding/folding, and immune response (IL1RN, ANXA11, SAMHD1) (75).

Microarray analysis of intracellular processes, induced early during differentiation of monocytes to vitD3-tolDC, revealed an upregulation of genes related to glucose metabolism, tricarboxylic acid cycle, and oxidative phosphorylation, including *GLU3, HK3, PFKFB4*, *PDHA1, LDHA, ATP5A1*, and the transcription factor *C-MYC* (34). Glucose availability and glycolysis, controlled by the PI3K/Akt/mTOR pathway, were shown to dictate the induction and maintenance of the tolerogenic phenotype and function in vitD3-modulated tolDCs (34).

Similar results were reported by Malinarich and colleagues, who compared transcriptomes of tolDCs modulated by dexamethasone and vitD3, with or without activation by LPS, to those of immature and LPS-matured DCs (74). This study confirmed the upregulation of catabolic pathways, including oxidative phosphorylation, fatty acid metabolism, and glycolysis, in vitD3-modulated tolDCs compared to immature DCs (74). However, LPS-induced activation was shown to decrease the metabolic plasticity in tolDCs and DCs, mainly by negatively regulating oxidative phosphorylation, without affecting mitochondrial function (74).

Using a different approach, Zimmer and colleagues analyzed proteomes of human moDCs, either modulated with dexamethasone or activated with LPS or peptidoglycan, using DIGE and label-free mass spectrometry to identify putative biomarkers of tolDCs (72). Proteomic analysis uncovered 14 potential marker candidates that were significantly upregulated in tolDCs compared to immature DCs and LPS- or peptidoglycan-matured DCs, including FKBP5, GPX1, C1QA, and STAB1 (72). Evaluation of candidate expression in other tolDC types, modulated by IL-10, rapamycin, vitD3, TGF-β, or *Aspergillus oryzae* protease, through qPCR and Western blot analysis, revealed substantial heterogenicity. Only ANXA1, CATC, and GILZ were upregulated in all tolDCs subtypes and therefore suggested as tolDC markers (72).

Different tolDC types share main phenotypic and functional characteristics, however, transcriptome and proteome studies demonstrated that each modulatory agent, used to promote tolDCs, induces a distinct transcriptional program in DCs (Table 1). While IL-10 mainly affects immunological processes (71), vitD3 has a major impact on metabolic pathways, involving oxidative phosphorylation, fatty acid, and glucose metabolism (34, 74). By contrast, dexamethasone exerts an influence on glutathione metabolism and upregulates genes related to stress response and redox homeostasis (72, 74, 75). There are only few common molecules found to be upregulated in different tolDC types, including the cytokine IL-1Ra (IL1RN), complement component 1q (C1Q), coagulation factor XIIIa (F13A), thrombospondin-1 (THBS1/TSP1), and superoxide dismutase (SOD2). IL-1Ra competes with IL-1 for binding to the IL-1 receptor, without inducing any intracellular response, and has been shown to inhibit DC maturation as well as T cell activation and polarization (76, 77). C1q was proposed to render DCs tolerogenic, by reducing the expression of co-stimulatory molecules and promoting high levels of immunosuppressive IL-10 and TGF-β (78, 79). F13A+ DCs were shown to produce retinoic acid and induce Foxp3+ Tregs (80). THBS1 is directly associated with tolerance induction, by impairing T effector cell proliferation while promoting Treg generation through ligation with its receptor CD47/IAP (69, 81, 82). Only SOD2 was found to be upregulated in all tolDC types described herein, irrespective of subsequent activation via TLR or CD40 (34, 69, 73, 75). This antioxidative enzyme is also expressed by immature and mature DCs (33, 36, 69), and is crucial for ­oxidative stress resistance and the regulation of inflammatory processes by attenuating the activity of NF-κB (83, 84). Accordingly, in mice with heterozygous SOD2 deficiency, DCs accumulate reactive oxygen species under stress conditions, secrete higher amounts of IL-6, CXCL1, and CXCL2/MIP-2α, and show an impaired antigen-presenting and co-stimulatory capacity, and decreased TNF-α secretion upon activation (85).

It is to be noted that the expression profiles of tolDCs show some overlap with those of immature DCs, e.g., upregulation of C/EBP, c-myc, p53, and SOD2 transcripts, which might be due to the inhibition of maturation/activation (34, 36). However, the transcriptome and proteome studies described herein unraveled distinctive molecular signatures of tolDCs, indicating that tolerogenic features emerge from a specific transcriptional program, rather than resulting from retention at an immature state.

## Concluding Remarks

Knowledge about molecular mechanisms that govern DC differentiation and function has increased due to technological advances. However, molecular switches that “turn on” tolerogenic functions in DCs remain largely unknown. Comparative transcriptome studies confirmed that tolDCs possess a characteristic molecular signature rather than being retained at a phenotypic and functional immature/semi-mature state. Since diverse modulating agents used for the generation of human tolDCs target distinct signaling pathways, the identification of master regulators of DC tolerogenicity has been challenging. Further comparative “omics” studies are required to define which molecules induce an immunoregulatory profile and thus might be used as targets to render DCs tolerogenic and to enhance their stability, longevity, and resistance to stress or pro-inflammatory stimuli for immunotherapeutic application.

## Conflict of Interest Statement

The authors declare that the research was conducted in the absence of any commercial or financial relationships that could be construed as a potential conflict of interest.
